# A New Positioning Method Based on Multiple Ultrasonic Sensors for Autonomous Mobile Robot

**DOI:** 10.3390/s20010017

**Published:** 2019-12-18

**Authors:** Mingqi Shen, Yuying Wang, Yandan Jiang, Haifeng Ji, Baoliang Wang, Zhiyao Huang

**Affiliations:** State Key Laboratory of Industrial Control Technology, College of Control Science and Engineering, Zhejiang University, Hangzhou 310027, China; shenmq@zju.edu.cn (M.S.); ydjiang@zju.edu.cn (Y.J.); hfji@zju.edu.cn (H.J.); wangbl@zju.edu.cn (B.W.)

**Keywords:** ultrasonic positioning, multiple ultrasonic sensors, autonomous mobile robot, sensor configuration, ratios of time-of-flights

## Abstract

This work proposes a new positioning method based on multiple ultrasonic sensors for the autonomous mobile robot. Unlike the conventional ultrasonic positioning methods, this new method can realize higher accuracy ultrasonic positioning without additional temperature information. Three ultrasonic sensors are used for positioning. A generalized measurement model is established for general sensor configuration. A simplified measurement model, which considers the computational complexity, is also established for linear/simplified sensor configuration. Three time-of-flight signals are obtained from the three ultrasonic sensors. The coordinates of the target are calculated by the ratios of time-of-flights. Positioning experiments were carried out to verify the feasibility and effectiveness of the proposed method. Experimental results show that the new ultrasonic positioning method is effective, both the two established models can implement positioning successfully, and the positioning accuracy is satisfactory. Compared with the conventional ultrasonic positioning method with the default ultrasonic velocity, the positioning accuracy is greatly improved by the proposed method. Compared with the ultrasonic positioning method with additional temperature compensation, the results obtained by the proposed method are comparable.

## 1. Introduction

With the development of computer science, automatic control technology, and artificial intelligence, the autonomous mobile robot has been widely used in industrial, logistic, and military fields. Positioning is one of the key techniques. Currently, optical methods and ultrasonic methods are the two main methods for positioning [1,2,3,4,5,6,7]. Compared with optical sensors, ultrasonic sensors have the advantages of low cost, low power consumption, and low computational complexity [8,9,10]. Furthermore, ultrasonic sensors can be used in opaque/non-transparent environments where optical sensors are difficult to work [11,12,13,14,15]. However, the measurement accuracy of ultrasonic sensors is not very high. The ultrasonic velocity is easily affected by environmental temperature. If the environmental temperature is unknown, a non-ignorable measurement error will occur in practical applications [16,17,18,19].

In the past several decades, much research has been undertaken to improve the accuracy of ultrasonic positioning systems based on temperature compensation methods [12,20,21,22,23,24,25,26,27]. The simplest method is to install a temperature sensor near the ultrasonic sensor [12]. According to the relationship between the ultrasonic velocity and temperature, the ultrasonic velocity can be compensated. Shinji and Junya proposed a measurement method for cross-sectional temperature and wind velocity distribution based on acoustic computer tomography [23]. Chande and Sharma proposed a compensation method based on the comparison of an unknown distance with a standard distance in the same medium [24]. Huang and Young presented an accurate ultrasonic distance measurement system which had a self-temperature compensation with the environmental average temperature in space [25]. Although these methods can improve the positioning performance, they need to introduce additional temperature sensors and a calibration process, which make the positioning system more complex.

This work aims to propose a new positioning method based on multiple ultrasonic sensors (three sensors in this work). The new method can implement higher accuracy ultrasonic positioning without additional temperature information or calibration process. The coordinates of the target are calculated according to the ratios of three time-of-flights measured by the three ultrasonic sensors, which means the influence of environmental temperature on positioning is avoided. Different from the previous/conventional methods using time-of-flights directly for positioning [12,20,21,22,23,24,25,26,27], this work features in a novel idea which uses the ratios of time-of-flights to realize ultrasonic positioning and hence to improve the positioning accuracy.

## 2. New Ultrasonic Positioning Method

**Figure 1:**
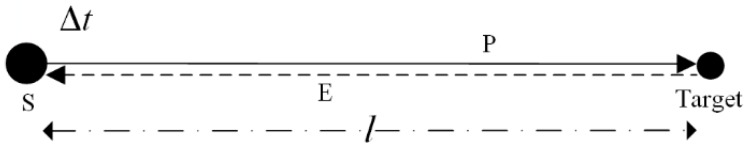
Measurement principle of ultrasonic ranging.

Figure 1 shows the basic measurement principle of ultrasonic ranging.

The ultrasonic sensor (S) serves as both the transmitter of an ultrasonic pulse (P) and receiver of an ultrasonic echo (E). The propagation time of the ultrasonic wave is called the time-of-flight (Δt) and is measured by comparing the ultrasonic pulse and echo. The distance (l) between the target and the ultrasonic sensor can be calculated by Equation (1) [28,29,30,31].

(1) $$l=\;\frac{c_{t}\Delta t}{2}$$

As we know, ultrasonic velocity $c_{t}$ is affected by environmental temperature. If the environmental temperature is variable and unknown, a non-ignorable measurement error will arise. If we can seek an approach to implement higher accuracy ultrasonic positioning without the need of ultrasonic velocity information, the influence of environmental temperature on ultrasonic positioning can be avoided. On the basis of this idea, a new ultrasonic positioning method is proposed and three ultrasonic sensors are used for positioning. The ratios of time-of-flights are first calculated with the time-of-flights obtained by the three ultrasonic sensors. On the basis of the ratios, the coordinates of the target (x,y) are calculated and hence positioning is implemented. In this way, no ultrasonic velocity information is needed and the influence of temperature on positioning is avoided. The main scheme of the proposed method includes two steps:

1. Modeling. A generalized measurement model is established for general sensor configuration (no special restriction/limitation on the position of the three ultrasonic sensors). Considering the computational complexity and practical measurement, a simplified measurement model is also established for simplified/linear sensor configuration.
2. Experiments. Actual positioning experiments are carried out to verify the feasibility and effectiveness of the established models.

### 2.1. Generalized Measurement Model

**Figure 2:**
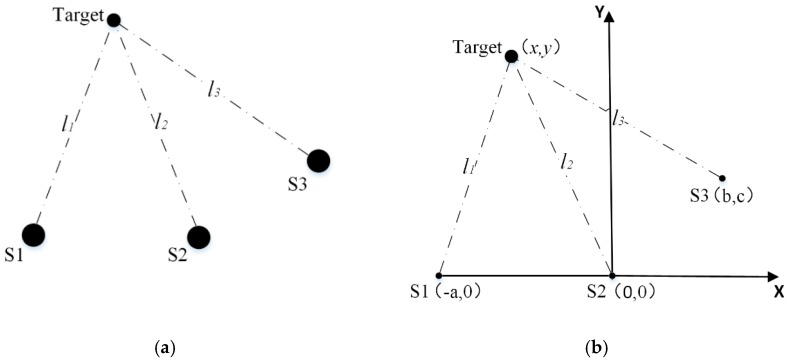
(**a**) General sensor configuration of the three ultrasonic sensors; (**b**) the coordinates of the general sensor configuration.

For the general sensor configuration, there is no special restriction/limitation on the position of the three ultrasonic sensors (i.e., the coordinates of the target (x,y) and the coordinates of the three ultrasonic sensors are freely configured), as shown in Figure 2, where S1, S2, and S3 are Sensor 1, Sensor 2, and Sensor 3.

Assuming the coordinates of S1, S2, and S3 are (−a, 0), (0, 0) and (b,c). The distances between the target and S1, S2, and S3 are $l_{1}$, $l_{2}$, and $l_{3}$ respectively. The corresponding time-of-flights of $l_{1}$, $l_{2}$, and $l_{3}$ are $\Delta t_{1}$, $\Delta t_{2}$, and $\Delta t_{3}$ respectively. According to Equation (1), the following relationships can be obtained:

(2) $$\{\begin{array}{c} \Delta t_{1}=\frac{2l_{1}}{c_{t}}\\ \Delta t_{2}=\frac{2l_{2}}{c_{t}}\\ \Delta t_{3}=\frac{2l_{3}}{c_{t}} \end{array}$$

In this work, the ratios of time-of-flights are used to calculate the coordinates of the target (x,y). Meanwhile, considering that the calculation error will be amplified when the denominator is too small, it is important to choose a suitable denominator for the ratios. Here, the maximum of the three time-of-flights is selected as the denominator. Assuming $\Delta t_{2}$ is the maximum of the three time-of-flights, let

(3) $$\{\begin{array}{c} \frac{\Delta t_{1}}{\Delta t_{2}}\;=k_{1}\\ \frac{\Delta t_{3}}{\Delta t_{2}}\;=k_{2} \end{array}$$

According to Figure 2, $k_{1}$ and $k_{2}$ could be rewritten as:

(4) $$\;k_{1}=\frac{\Delta t_{1}}{\Delta t_{2}}=\frac{l_{1}}{l_{2}}=\frac{\sqrt{{\left(x+a\right)}^{2}+y^{2}}}{\sqrt{x^{2}+y^{2}}}\;$$

(5) $$k_{2}=\frac{\Delta t_{3}}{\Delta t_{2}}=\frac{l_{3}}{l_{2}}=\frac{\sqrt{{\left(x-b\right)}^{2}+{\left(y-c\right)}^{2}}}{\sqrt{x^{2}+y^{2}}}$$

Further, Equations (4) and (5) could be rewritten as:

(6) $$k_{1}\sqrt{x^{2}+y^{2}}=\;\sqrt{{\left(x+a\right)}^{2}+y^{2}}$$

(7) $$k_{2}\sqrt{x^{2}+y^{2}}\;=\;\sqrt{{\left(x-b\right)}^{2}+{\left(y-c\right)}^{2}}$$

Squaring both sides of Equations (6) and (7), we can get the following equations:

(8) $$\left(1-k_{1}{}^{2}\right)x^{2}+2ax+a^{2}+\left(1-k_{1}{}^{2}\right)y^{2}=0$$

(9) $$\left(1-k_{2}{}^{2}\right)x^{2}-2bx+b^{2}+\left(1-k_{2}{}^{2}\right)y^{2}-2cy+c^{2}=0$$

The coordinates of the target (x,y) are the solutions of Equations (8) and (9). Obviously, there are two independent equations and two unknowns (x and y), so the solutions exist. That means with the ratios of time-of-flights, the coordinates of the target (x,y) can be determined without ultrasonic velocity information.

In detail, there are four different cases (with different values of $k_{1}$ and $k_{2}$), and the four measurement models are developed as follows:

(1) For $k_{1}=\;1$ and $k_{2}=\;1$, Equations (8) and (9) can be rewritten as:

(10) $$\{\begin{array}{c} 2ax+a^{2}=0\\ -2bx-2cy+b^{2}+c^{2}=0 \end{array}$$

The coordinates of the target (x,y) are the intersection of two lines, and the values of *x* and *y* can be obtained as:

(11) $$\{\begin{array}{c} x=-\frac{a}{2}\\ y=\;\frac{ab+b^{2}+c^{2}}{2c} \end{array}$$

(2) For $k_{1}=\;1$ and $k_{2}\neq \;1$, Equations (8) and (9) can be rewritten as:

(12) $$\{\begin{array}{c} 2ax+a^{2}=0\\ {\left(x-\frac{b}{1-k_{2}{}^{2}}\right)}^{2}+{\left(y-\frac{c}{1-k_{2}{}^{2}}\right)}^{2}={\left(\frac{k_{2}}{1-k_{2}{}^{2}}\right)}^{2}\left(b^{2}+c^{2}\right)\;k_{2}=\;\frac{\Delta t_{3}}{\Delta t_{2}} \end{array}$$

The coordinates of the target (x,y) are the two intersections of a line and a circle. Correspondingly, two sets of values for x and y can be obtained as:

(13) $$\{\begin{array}{c} x_{1}=-\frac{a}{2}\\ y_{1}=\;\frac{c\Delta t_{2}{}^{2}}{\Delta t_{2}{}^{2}-\Delta t_{3}{}^{2}}+\sqrt{{\left(\frac{\Delta t_{2}\Delta t_{3}}{\Delta t_{2}{}^{2}-\Delta t_{3}{}^{2}}\right)}^{2}\left(b^{2}+c^{2}\right)-{\left(\frac{a}{2}-\frac{b\Delta t_{2}{}^{2}}{\Delta t_{2}{}^{2}-\Delta t_{3}{}^{2}}\right)}^{2}} \end{array}$$

(14) $$\{\begin{array}{c} x_{2}=-\frac{a}{2}\\ y_{2}=\;\frac{c\Delta t_{2}{}^{2}}{\Delta t_{2}{}^{2}-\Delta t_{3}{}^{2}}-\sqrt{{\left(\frac{\Delta t_{2}\Delta t_{3}}{\Delta t_{2}{}^{2}-\Delta t_{3}{}^{2}}\right)}^{2}\left(b^{2}+c^{2}\right)-{\left(\frac{a}{2}-\frac{b\Delta t_{2}{}^{2}}{\Delta t_{2}{}^{2}-\Delta t_{3}{}^{2}}\right)}^{2}} \end{array}$$

(3) For $k_{1}\neq \;1$ and $k_{2}=\;1$, Equations (8) and (9) can be rewritten as:

(15) $$\{\begin{array}{c} {\left(x+\frac{a}{1-k_{1}{}^{2}}\right)}^{2}+y^{2}={\left(\frac{ak_{1}}{1-k_{1}{}^{2}}\right)}^{2}\;k_{1}=\;\frac{\Delta t_{1}}{\Delta t_{2}}\\ -2bx-2cy+b^{2}+c^{2}=0 \end{array}$$

The coordinates of the target (x,y) are the two intersections of a circle and a line. Correspondingly, two sets of values for x and y can be obtained as:

(16) $$\{\begin{array}{c} x_{1}=\frac{b}{2}+\frac{-\frac{2ac^{2}\Delta t_{2}{}^{2}}{\Delta t_{2}{}^{2}-\Delta t_{1}{}^{2}}+c^{2}\sqrt{U}}{2\left(b^{2}+c^{2}\right)}\\ y_{1}=\;\frac{c}{2}+\frac{\frac{2abc\Delta t_{2}{}^{2}}{\Delta t_{2}{}^{2}-\Delta t_{1}{}^{2}}-bc\sqrt{U}}{2\left(b^{2}+c^{2}\right)}\; \end{array}$$

(17) $$\{\begin{array}{c} x_{2}=\frac{b}{2}+\frac{-\frac{2ac^{2}\Delta t_{2}{}^{2}}{\Delta t_{2}{}^{2}-\Delta t_{1}{}^{2}}-c^{2}\sqrt{U}}{2\left(b^{2}+c^{2}\right)}\\ y_{2}=\;\frac{c}{2}+\frac{\frac{2abc\Delta t_{2}{}^{2}}{\Delta t_{2}{}^{2}-\Delta t_{1}{}^{2}}+bc\sqrt{U}}{2\left(b^{2}+c^{2}\right)}\; \end{array}$$

where

(18) $$U\;=\;{\left[\frac{2a\Delta t_{2}{}^{2}}{\Delta t_{2}{}^{2}-\Delta t_{1}{}^{2}}-\frac{b\left(b^{2}+c^{2}\right)}{c^{2}}\right]}^{2}-4\left(\frac{b^{2}+c^{2}}{c^{2}}\right)\left[\frac{a^{2}\Delta t_{2}{}^{2}}{\Delta t_{2}{}^{2}-\Delta t_{1}{}^{2}}+\frac{{\left(b^{2}+c^{2}\right)}^{2}}{4c^{2}}\right]$$

(4) For $k_{1}\neq \;1$ and $k_{2}\neq \;1$, Equations (8) and (9) can be rewritten as:

(19) $$\{\begin{array}{c} {\left(x+\frac{a}{1-k_{1}{}^{2}}\right)}^{2}+y^{2}={\left(\frac{ak_{1}}{1-k_{1}{}^{2}}\right)}^{2}\;k_{1}=\;\frac{\Delta t_{1}}{\Delta t_{2}}\\ {\left(x-\frac{b}{1-k_{2}{}^{2}}\right)}^{2}+{\left(y-\frac{c}{1-k_{2}{}^{2}}\right)}^{2}={\left(\frac{k_{2}}{1-k_{2}{}^{2}}\right)}^{2}\left(b^{2}+c^{2}\right)\;k_{2}=\;\frac{\Delta t_{3}}{\Delta t_{2}} \end{array}\;$$

The coordinates of the target (x,y) are the two intersections of two circles. Let

(20) $$\{\begin{array}{c} p_{1}=\frac{-a}{1-k_{1}{}^{2}},p_{2}=\frac{b}{1-k_{2}{}^{2}},p_{3}=\frac{c}{1-k_{2}{}^{2}}\\ r_{1}^{2}={\left(\frac{ak_{1}}{1-k_{1}{}^{2}}\right)}^{2},r_{2}^{2}={\left(\frac{k_{2}}{1-k_{2}{}^{2}}\right)}^{2}\left(b^{2}+c^{2}\right)\\ D=\sqrt{p_{3}{}^{2}+{\left(p_{2}-p_{1}\right)}^{2}} \end{array}$$

With Equation (19) and (20), two corresponding sets of values for x and y can be obtained as:

(21) $$\{\begin{array}{c} x_{1}=p_{1}+\frac{r_{1}^{2}-r_{2}^{2}+D^{2}}{2D^{2}}\left(p_{2}-p_{1}\right)-p_{3}\frac{\sqrt{4r_{1}^{2}D^{2}-{\left(r_{1}^{2}-r_{2}^{2}+D^{2}\right)}^{2}}}{2D^{2}}\\ y_{1}=\frac{r_{1}^{2}-r_{2}^{2}+D^{2}}{2D^{2}}p_{3}+\left(p_{2}-p_{1}\right)\frac{\sqrt{4r_{1}^{2}D^{2}-{\left(r_{1}^{2}-r_{2}^{2}+D^{2}\right)}^{2}}}{2D^{2}} \end{array}$$

(22) $$\{\begin{array}{c} x_{2}=p_{1}+\frac{r_{1}^{2}-r_{2}^{2}+D^{2}}{2D^{2}}\left(p_{2}-p_{1}\right)+p_{3}\frac{\sqrt{4r_{1}^{2}D^{2}-{\left(r_{1}^{2}-r_{2}^{2}+D^{2}\right)}^{2}}}{2D^{2}}\\ y_{2}=\frac{r_{1}^{2}-r_{2}^{2}+D^{2}}{2D^{2}}p_{3}-\left(p_{2}-p_{1}\right)\frac{\sqrt{4r_{1}^{2}D^{2}-{\left(r_{1}^{2}-r_{2}^{2}+D^{2}\right)}^{2}}}{2D^{2}} \end{array}$$

Among the above four cases, only the first case has a unique solution, the rest three cases all have two solutions. In cases (2), (3), and (4), it is necessary to identify which set of (x,y) is the actual coordinates. As we know, the ultrasonic velocity has a reasonable range under known circumstance. In this work, for each set of coordinates (x,y), a corresponding ultrasonic velocity could be calculated. If one of the ultrasonic velocities is in the reasonable range, the corresponding set of (x,y) is the actual coordinates of the target. Usually, the ultrasonic velocity corresponding to the other set of (x,y) is out of the reasonable range and can be excluded.

It is necessary to indicate that for case (2), (3) and (4), there exist special conditions where the ultrasonic velocities corresponding to the two sets of (x,y) are both in the reasonable range. Theoretically, the special condition can be identified by a coefficient ε. If the value of ε is close to zero (ε→0), the ultrasonic velocities corresponding to the two sets of (x,y) will be both in the reasonable range. The detailed descriptions of the ε are listed as follows:

For case (2),

(23) $$\varepsilon =\left|{\left(\frac{\Delta t_{2}\Delta t_{3}}{\Delta t_{2}{}^{2}-\Delta t_{3}{}^{2}}\right)}^{2}\left(b^{2}+c^{2}\right)-{\left(\frac{a}{2}-\frac{b\Delta t_{2}{}^{2}}{\Delta t_{2}{}^{2}-\Delta t_{3}{}^{2}}\right)}^{2}\right|$$

For case (3),

(24) $$\varepsilon =\left|{\left[\frac{2a\Delta t_{2}{}^{2}}{\Delta t_{2}{}^{2}-\Delta t_{1}{}^{2}}-\frac{b\left(b^{2}+c^{2}\right)}{c^{2}}\right]}^{2}-4\left(\frac{b^{2}+c^{2}}{c^{2}}\right)\left[\frac{a^{2}\Delta t_{2}{}^{2}}{\Delta t_{2}{}^{2}-\Delta t_{1}{}^{2}}+\frac{{\left(b^{2}+c^{2}\right)}^{2}}{4c^{2}}\right]\right|$$

For case (4),

(25) $$\varepsilon =\left|4r_{1}^{2}D^{2}-{\left(r_{1}^{2}-r_{2}^{2}+D^{2}\right)}^{2}\right|$$

where $r_{1}$, $r_{2}$ and D can be obtained by Equation (20).

In the practical positioning process, for the above special conditions, there are two ways to determine the actual coordinates (x,y), introducing an extra fourth ultrasonic sensor or making a second positioning by changing the coordinates of the three ultrasonic sensors (e.g., moving the location of the mobile robot). The latter approach is preferred here because it can be conveniently implemented with the same sensor configuration. In this way, two new sets of (x,y) can be obtained and the actual coordinates of the target can be determined by choosing the common set of (x,y) among the positioning results before and after the moving adjustment. Detailed examples of the identifying process can be found in Section 3.2: Experimental Results.

### 2.2. Simplified Measurement Model

**Figure 3:**
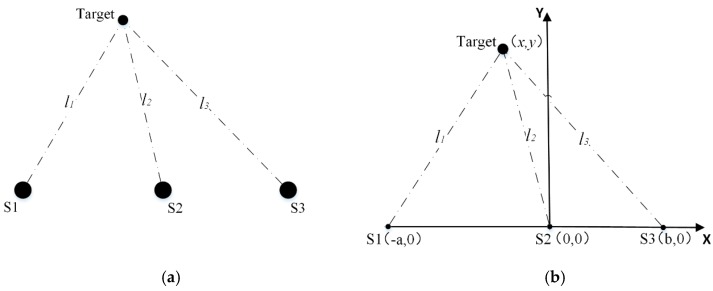
(**a**) Simplified sensor configuration of the three ultrasonic sensors; (**b**) the coordinates of the simplified sensor configuration.

The simplified sensor configuration (i.e., the three ultrasonic sensors are configured in a straight line) is usually adopted to reduce the complexity and simplify the measurement, as shown in Figure 3.

Assuming the coordinates of the three ultrasonic sensors are S1(−a, 0), S2(0, 0), and S3(b, 0) correspondingly. Similar to the determination of Equations (8) and (9), we can get the following equations:

(26) $$\left(1-k_{1}{}^{2}\right)x^{2}+2ax+a^{2}+\left(1-k_{1}{}^{2}\right)y^{2}=0$$

(27) $$\left(1-k_{2}{}^{2}\right)x^{2}-2bx+b^{2}+\left(1-k_{2}{}^{2}\right)y^{2}=0$$

The coordinates of the target (x,y) are the solutions of Equations (26) and (27). In detail, there are three different cases (with different values of $k_{1}$ and $k_{2}$), and three measurement models are developed as follows:

(1) For $k_{1}=\;1$ and $k_{2}\neq \;1$, Equations (26) and (27) can be rewritten as:

(28) $$\{\begin{array}{c} 2ax+a^{2}=0\\ {\left(x-\frac{b}{1-k_{2}{}^{2}}\right)}^{2}+y^{2}={\left(\frac{bk_{2}}{1-k_{2}{}^{2}}\right)}^{2}\;k_{2}=\;\frac{\Delta t_{3}}{\Delta t_{2}} \end{array}$$

The coordinates of the target (x,y) are the two intersections of a line and a circle. Correspondingly, two sets of values for x and y can be obtained as:

(29) $$\{\begin{array}{c} x_{1}=-\frac{a}{2}\\ y_{1}=\sqrt{\frac{\left(a+b\right)b\cdot \Delta t_{2}{}^{2}}{\Delta t_{3}{}^{2}-\Delta t_{2}{}^{2}}-\frac{a^{2}}{4}} \end{array}$$

(30) $$\{\begin{array}{c} x_{2}=-\frac{a}{2}\\ y_{2}=-\sqrt{\frac{\left(a+b\right)b\cdot \Delta t_{2}{}^{2}}{\Delta t_{3}{}^{2}-\Delta t_{2}{}^{2}}-\frac{a^{2}}{4}} \end{array}$$

(2) For $k_{1}\neq \;1$ and $k_{2}=\;1$, Equations (26) and (27) can be rewritten as:

(31) $$\{\begin{array}{c} {\left(x+\frac{a}{1-k_{1}{}^{2}}\right)}^{2}+y^{2}={\left(\frac{ak_{1}}{1-k_{1}{}^{2}}\right)}^{2}\;k_{1}=\;\frac{\Delta t_{1}}{\Delta t_{2}}\\ -2bx+b^{2}=0 \end{array}$$

The coordinates of the target (x,y) are the two intersections of a circle and a line. Correspondingly, two sets of values for x and y can be obtained as:

(32) $$\{\begin{array}{c} x_{1}=\frac{b}{2}\\ y_{1}=\sqrt{\frac{\left(a+b\right)a\cdot \Delta t_{2}{}^{2}}{\Delta t_{1}{}^{2}-\Delta t_{2}{}^{2}}-\frac{b^{2}}{4}} \end{array}$$

(33) $$\{\begin{array}{c} x_{2}=\frac{b}{2}\\ y_{2}=-\sqrt{\frac{\left(a+b\right)a\cdot \Delta t_{2}{}^{2}}{\Delta t_{1}{}^{2}-\Delta t_{2}{}^{2}}-\frac{b^{2}}{4}} \end{array}\;$$

(3) For $k_{1}\neq \;1$ and $k_{2}\neq \;1$, Equations (26) and (27) can be rewritten as:

(34) $$\{\begin{array}{c} {\left(x+\frac{a}{1-k_{1}{}^{2}}\right)}^{2}+y^{2}={\left(\frac{ak_{1}}{1-k_{1}{}^{2}}\right)}^{2}\;k_{1}=\;\frac{\Delta t_{1}}{\Delta t_{2}}\\ {\left(x-\frac{b}{1-k_{2}{}^{2}}\right)}^{2}+y^{2}={\left(\frac{bk_{2}}{1-k_{2}{}^{2}}\right)}^{2}\;k_{2}=\;\frac{\Delta t_{3}}{\Delta t_{2}} \end{array}$$

The coordinates of the target (x,y) are the two intersections of two circles. Correspondingly, two sets of values for x and y can be obtained as:

(35) $$\{\begin{array}{c} x_{1}=\frac{a^{2}\left(\Delta t_{3}{}^{2}-\Delta t_{2}{}^{2}\right)-b^{2}\left(\Delta t_{1}{}^{2}-\Delta t_{2}{}^{2}\right)}{2a\left(\Delta t_{2}{}^{2}-\Delta t_{3}{}^{2}\right)+2b\left(\Delta t_{2}{}^{2}-\Delta t_{1}{}^{2}\right)}\\ y_{1}=\sqrt{\frac{\left(a^{2}b+ab^{2}\right)\Delta t_{2}{}^{2}}{a\left(\Delta t_{3}{}^{2}-\Delta t_{2}{}^{2}\right)+b\left(\Delta t_{1}{}^{2}-\Delta t_{2}{}^{2}\right)}-{\left[\frac{a^{2}\left(\Delta t_{3}{}^{2}-\Delta t_{2}{}^{2}\right)-b^{2}\left(\Delta t_{1}{}^{2}-\Delta t_{2}{}^{2}\right)}{2a\left(\Delta t_{2}{}^{2}-\Delta t_{3}{}^{2}\right)+2b\left(\Delta t_{2}{}^{2}-\Delta t_{1}{}^{2}\right)}\right]}^{2}} \end{array}$$

(36) $$\{\begin{array}{c} x_{2}=\frac{a^{2}\left(\Delta t_{3}{}^{2}-\Delta t_{2}{}^{2}\right)-b^{2}\left(\Delta t_{1}{}^{2}-\Delta t_{2}{}^{2}\right)}{2a\left(\Delta t_{2}{}^{2}-\Delta t_{3}{}^{2}\right)+2b\left(\Delta t_{2}{}^{2}-\Delta t_{1}{}^{2}\right)}\\ y_{2}=-\sqrt{\frac{\left(a^{2}b+ab^{2}\right)\Delta t_{2}{}^{2}}{a\left(\Delta t_{3}{}^{2}-\Delta t_{2}{}^{2}\right)+b\left(\Delta t_{1}{}^{2}-\Delta t_{2}{}^{2}\right)}-{\left[\frac{a^{2}\left(\Delta t_{3}{}^{2}-\Delta t_{2}{}^{2}\right)-b^{2}\left(\Delta t_{1}{}^{2}-\Delta t_{2}{}^{2}\right)}{2a\left(\Delta t_{2}{}^{2}-\Delta t_{3}{}^{2}\right)+2b\left(\Delta t_{2}{}^{2}-\Delta t_{1}{}^{2}\right)}\right]}^{2}} \end{array}$$

All the above three cases have two solutions. It is necessary to identify which set of (x,y) is the actual coordinates. In this work, according to the sensor configuration, the value of y must be positive, so the set with negative value of y can be excluded. Obviously, Equations (29), (32), and (35) are the actual coordinates of the target corresponding to the three cases. In the simplified configuration, the computational complexity is lower and an additional solution identifying process needed in the generalized measurement model can be avoided.

## 3. Experimental Results

### 3.1. Experimental Setup

As the frequency of the ultrasonic sensor increases, its directivity will be better but the range of the measurable distance will decrease [11]. Considering the accuracy and measurable distance of the system, the ultrasonic sensor DYA-100-03H from Hangzhou Xingbao Electronic Technology Co., Ltd., Hangzhou, China, is selected and the frequency is 100 kHz.

**Figure 4:**
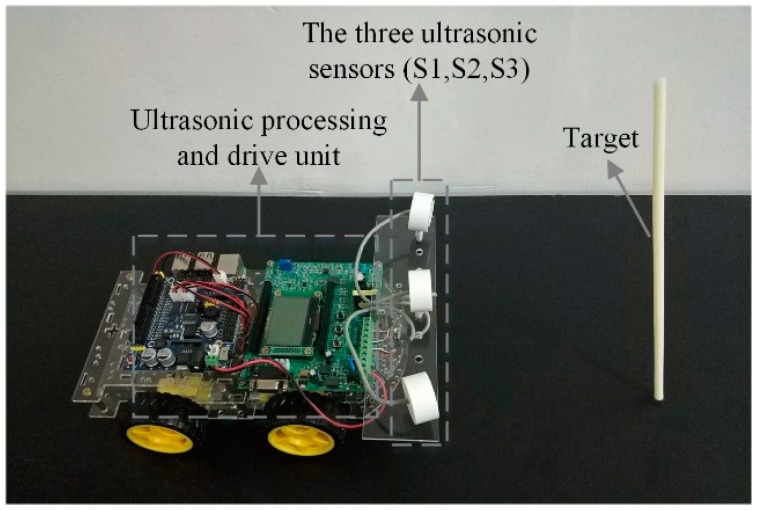
The photo of the experimental setup.

In order to meet the configuration requirements of the proposed method, a plastic rod was used as the experimental object (i.e., the target). The diameter of the plastic rod used is 6.0 mm, which is small enough when compared with the measured distance (200.0–600.0 mm). Therefore, this plastic rod was regarded as a point target in this work. Figure 4 is a photo of the experimental setup.

### 3.2. Experimental Results

In order to verify the effectiveness of the proposed method, the general sensor configuration in Figure 2 and the simplified sensor configuration in Figure 3 are constructed respectively and the corresponding experiments are carried out. The distances $l_{1}$, $l_{2}$, and $l_{3}$ between the target and S1, S2, and S3 are selected as the indicators to evaluate the positioning accuracy.

Define the distances $l_{1}$, $l_{2}$, and $l_{3}$ measured by the proposed method as $l_{m1}$, $l_{m2}$, and $l_{m3}$. According to the generalized measurement model, the three distances $l_{m1}$, $l_{m2}$, and $l_{m3}$ can be calculated as:

(37) $$\{\begin{array}{c} l_{m1}=\sqrt{{\left(x+a\right)}^{2}+y^{2}}\\ l_{m2}=\sqrt{x^{2}+y^{2}}\\ l_{m3}=\sqrt{{\left(x-b\right)}^{2}+{\left(y-c\right)}^{2}} \end{array}$$

where a,b,c is given, and x,y are determined with the generalized measurement model.

Similarly, according to the simplified measurement model, the distances $l_{m1}$, $l_{m2}$, and $l_{m3}$ can be calculated as:

(38) $$\{\begin{array}{c} l_{m1}=\sqrt{{\left(x+a\right)}^{2}+y^{2}}\\ l_{m2}=\sqrt{x^{2}+y^{2}}\\ l_{m3}=\sqrt{{\left(x-b\right)}^{2}+y^{2}} \end{array}$$

where a,b is given, and x,y are determined with the simplified measurement model.

In comparison, the reference distances (defined as $l_{r1}$, $l_{r2}$, $l_{r3}$) and the distances obtained by two conventional ultrasonic positioning methods (defined as $l_{d1}$, $l_{d2}$, $l_{d3}$ and $l_{c1}$, $l_{c2}$, $l_{c3}$ respectively) are also provided. The actual/reference distances $l_{r1}$, $l_{r2}$, and $l_{r3}$ are measured by tapeline. The distances $l_{d1}$, $l_{d2}$, and $l_{d3}$ are obtained by the conventional ultrasonic positioning method with default ultrasonic velocity. In this work, the default ultrasonic velocity is 340 m/s. The distances $l_{c1}$, $l_{c2},$ and $l_{c3}$ are obtained by the ultrasonic positioning method with temperature compensation at the experimental temperature. In this work, the experimental temperature is 25 °C.

The relative error of the distance measurement is introduced to show the measurement accuracy of the three methods, which is defined as:

(39) $$e=\;\frac{\left|l-l_{r}\right|}{l_{r}}\times 100\%$$

where l is the measured distance between the target and the sensor (i.e., $l_{d}$, $l_{c}$, or $l_{m}$) and $l_{r}$ is the reference distance between the target and the sensor.

#### 3.2.1. Experiment for the Generalized Measurement Model

**Figure 5:**
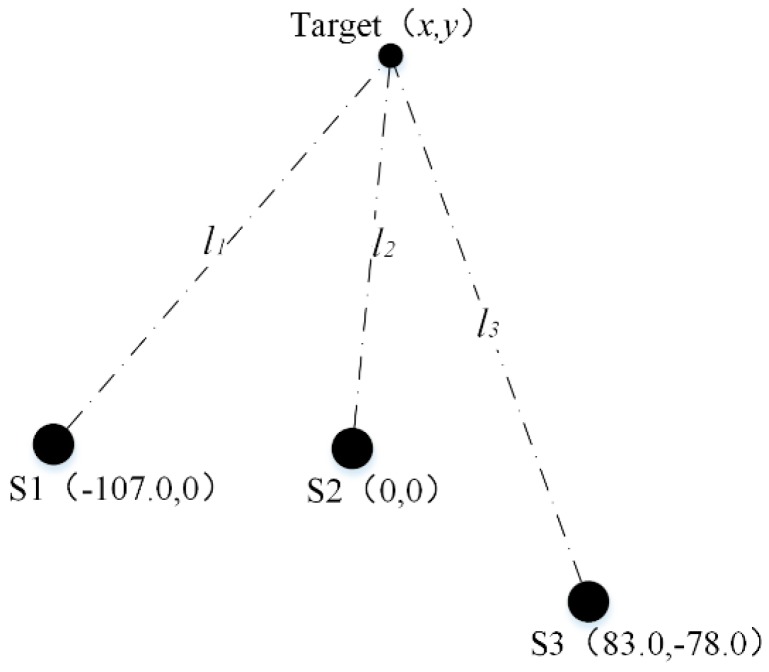
Schematic diagram of the experiment with general sensor configuration.

(1) The positioning experiment with the general sensor configuration was carried out with the parameter setup of a=107.0 mm, b=83.0 mm, c=−78.0 mm and with the experimental temperature of 25 °C. The schematic diagram of the experiment with general sensor configuration is shown in Figure 5.

The experimental results are shown in Table 1.

Taking the data of group 1 as an example to show the process of calculation and identification.

(40) $$\{\begin{array}{c} p_{1}=\frac{-a}{1-k_{1}{}^{2}}=113.8\;\mathrm{mm},p_{2}=\frac{b}{1-k_{2}{}^{2}}=-108.1\;\mathrm{mm},p_{3}=\frac{c}{1-k_{2}{}^{2}}=101.6\;\mathrm{mm}\\ r_{1}^{2}={\left(\frac{ak_{1}}{1-k_{1}{}^{2}}\right)}^{2}=25111.7\;{\mathrm{mm}}^{2},r_{2}^{2}={\left(\frac{k_{2}}{1-k_{2}{}^{2}}\right)}^{2}\left(b^{2}+c^{2}\right)=38925.0\;{\mathrm{mm}}^{2}\\ D=\sqrt{p_{3}{}^{2}+{\left(p_{2}-p_{1}\right)}^{2}}=244.1\;\mathrm{mm} \end{array}$$

(41) $$\{\begin{array}{c} x_{1}=p_{1}+\frac{r_{1}^{2}-r_{2}^{2}+D^{2}}{2D^{2}}\left(p_{2}-p_{1}\right)-p_{3}\frac{\sqrt{4r_{1}^{2}D^{2}-{\left(r_{1}^{2}-r_{2}^{2}+D^{2}\right)}^{2}}}{2D^{2}}=-24.7\;\mathrm{mm}\\ y_{1}=\frac{r_{1}^{2}-r_{2}^{2}+D^{2}}{2D^{2}}p_{3}+\left(p_{2}-p_{1}\right)\frac{\sqrt{4r_{1}^{2}D^{2}-{\left(r_{1}^{2}-r_{2}^{2}+D^{2}\right)}^{2}}}{2D^{2}}=-77.1\;\mathrm{mm} \end{array}$$

(42) $$\{\begin{array}{c} x_{2}=p_{1}+\frac{r_{1}^{2}-r_{2}^{2}+D^{2}}{2D^{2}}\left(p_{2}-p_{1}\right)+p_{3}\frac{\sqrt{4r_{1}^{2}D^{2}-{\left(r_{1}^{2}-r_{2}^{2}+D^{2}\right)}^{2}}}{2D^{2}}=81.7\;\mathrm{mm}\\ y_{2}=\frac{r_{1}^{2}-r_{2}^{2}+D^{2}}{2D^{2}}p_{3}-\left(p_{2}-p_{1}\right)\frac{\sqrt{4r_{1}^{2}D^{2}-{\left(r_{1}^{2}-r_{2}^{2}+D^{2}\right)}^{2}}}{2D^{2}}=155.2\;\mathrm{mm} \end{array}$$

(43) $$c_{t}=\frac{\sqrt{x_{1}{}^{2}+y_{1}{}^{2}}}{\Delta t_{2}}=159.2\;m/s$$

(44) $$c_{t}=\frac{\sqrt{x_{2}{}^{2}+y_{2}{}^{2}}}{\Delta t_{2}}=344.7\;m/s$$

For the common environmental temperature in the research field of ranging/positioning (0–45 °C), the reasonable range of ultrasonic velocity in air is about 330 m/s to 360 m/s [27]. According to the results in Equations (43) and (44), $\left(x_{1},y_{1}\right)$ can be excluded, and $\left(x_{2},y_{2}\right)$ is the actual coordinates of the target. As the values of x and y are determined, $l_{m1}$, $l_{m2}$, and $l_{m3}$ can be obtained by Equation (45).

(45) $$\{\begin{array}{c} l_{m1}=\sqrt{{\left(x+a\right)}^{2}+y^{2}}=244.4\;\mathrm{mm}\\ l_{m2}=\sqrt{x^{2}+y^{2}}=175.4\;\mathrm{mm}\\ l_{m3}=\sqrt{{\left(x-b\right)}^{2}+{\left(y-c\right)}^{2}}=233.2\;\mathrm{mm} \end{array}$$

As shown in Table 1, the positioning accuracy with the generalized measurement model is greatly improved, compared with the conventional ultrasonic positioning method with default ultrasonic velocity, and compared with that obtained by the positioning method with temperature compensation, the positioning result with the generalized measurement model is comparable, and has the advantage of no additional temperature sensors required.

**Figure 6:**
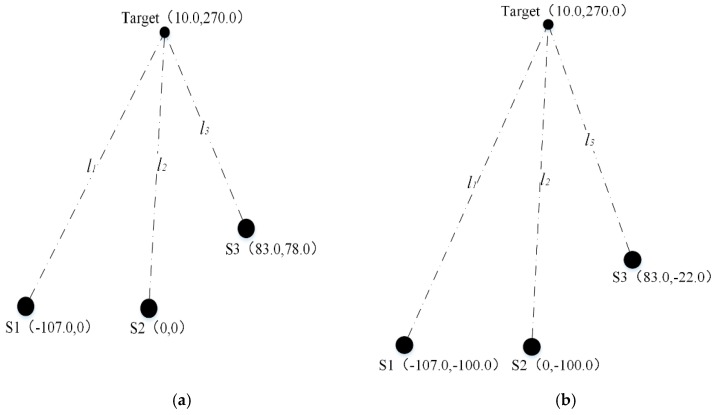
(**a**) Schematic diagram of the sensor configuration; (**b**) schematic diagram of the sensor configuration after moving adjustment.

(2) For the aforementioned special condition, where the ultrasonic velocities corresponding to the two sets of (x,y) are both in the reasonable range, the identifying process is explained through simulation experiments. The corresponding schematic diagram of the special sensor configuration with the parameter setup of a=107.0 mm, b=83.0 mm, c=78.0 mm is shown in Figure 6a.

Similar to the calculation of Equations (41) and (42), we can get the following equations:

(46) $$\{\begin{array}{c} x_{1}=10.0\;\mathrm{mm}\\ y_{1}=270.0\;\mathrm{mm} \end{array}$$

(47) $$\{\begin{array}{c} x_{2}=14.8\;\mathrm{mm}\\ y_{2}=279.8\;\mathrm{mm} \end{array}$$

(48) $$c_{0}=\frac{\sqrt{x_{1}{}^{2}+y_{1}{}^{2}}}{\Delta t_{2}}=346.0\;m/s$$

(49) $$c_{0}=\frac{\sqrt{x_{2}{}^{2}+y_{2}{}^{2}}}{\Delta t_{2}}=358.8\;m/s$$

As shown in Equations (48) and (49), the ultrasonic velocities corresponding to the two sets of (x,y) are both in the reasonable range, which means the actual set cannot be identified. Hence, positioning is implemented again after moving the coordinates of the three ultrasonic sensors along the Y- axis by −100 mm. The corresponding schematic diagram of the sensor configuration is shown in Figure 6b. Similarly, we can get the following equations:

(50) $$\{\begin{array}{c} x_{1}=-28.4\;\mathrm{mm}\\ y_{1}=131.0\;\mathrm{mm} \end{array}$$

(51) $$\{\begin{array}{c} x_{2}=10.0\;\mathrm{mm}\\ y_{2}=270.0\;\mathrm{mm} \end{array}$$

From Equations (46) and (47) and Equations (50) and (51), it is clear that (x,y)=(10.0,270.0) is the actual coordinates of the target.

#### 3.2.2. Experiment for the Simplified Measurement Model

**Figure 7:**
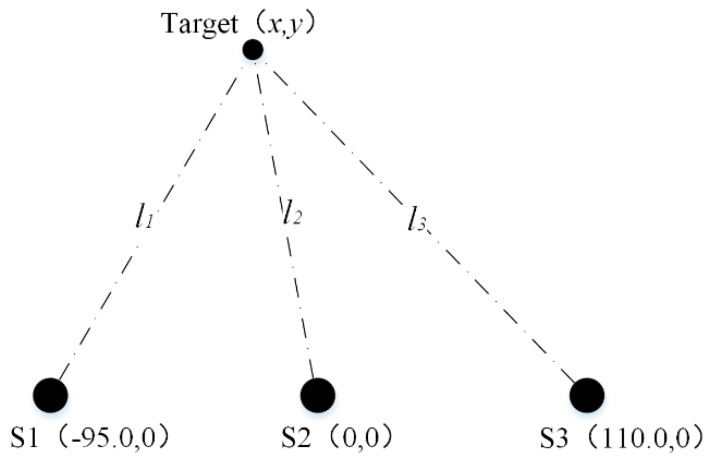
Schematic diagram of the experiment with simplified sensor configuration.

The positioning experiment with the simplified measurement model was carried out with the parameter setup of a=95.0 mm, b=110.0 mm and with the experimental temperature of 25 °C. Schematic diagram of the experiment with simplified sensor configuration is shown in Figure 7.

Similarly, the experimental results are shown in Table 2.

As shown in Table 2, the positioning accuracy with the simplified measurement model is greatly improved, compared with the conventional ultrasonic positioning method with default ultrasonic velocity, and compared with that obtained by the positioning method with temperature compensation, the positioning result with the simplified measurement model is comparable, and has the advantage of no additional temperature sensors required.

## 4. Discussion

This work investigates high accuracy positioning with both the general senor configuration and the simplified sensor configuration based on the three ultrasonic sensors. Correspondingly, two measurement models are established to implement positioning.

For the generalized measurement model, there are four different cases and three of them have two solutions. In this work, the identifying process is realized by checking whether the ultrasonic velocity corresponding to each set of (x,y) is in the reasonable range or not. In this way, the determination of (x,y) can be implemented for most cases. However, for special conditions where the ultrasonic velocities corresponding to the two sets of (x,y) are both in the reasonable range, the coordinates (x,y) are determined here by measuring again after moving the current configuration of the three sensors along the Y- axis. In detail, the actual coordinates of the target can be determined by choosing the common set of (x,y) among the positioning results before and after the moving adjustment. The feasibility of this adjustment is verified by an example of the special condition, as shown in Equations (46)–(51).

For the simplified measurement model, there are three different cases. Although all of them also have two solutions, the actual coordinates can be easily determined. In this work, according to the sensor configuration, the coordinates of the target (x,y) must have a positive y value, which means the actual set of (x,y) can be determined by excluding the set of (x,y) with negative y value. Compared with the generalized measurement model, the identifying process of the simplified measurement model is simpler and more convenient. The computational complexity of the simplified measurement model is lower and the complex solution identifying process needed in the generalized measurement model can be avoided. Hence, the simplified measurement model is recommended for practical applications.

## 5. Conclusions

This work proposes a new positioning method based on multiple ultrasonic sensors for the autonomous mobile robot. Without additional temperature sensors, three ultrasonic sensors are used for positioning. A generalized measurement model is established for general sensor configuration. A simplified measurement model, which considers the computational complexity, is also established for simplified/linear sensor configuration. Three time-of-flights signals are obtained from the three ultrasonic sensors. The coordinates of the target are calculated according to the ratios of time-of-flights.

The practical ultrasonic positioning experiments were carried out to verify the feasibility and effectiveness of the proposed method. Experimental results show that the new ultrasonic positioning method is effective, both the two established models can implement positioning successfully, and the positioning accuracy is satisfactory. Compared with the conventional ultrasonic positioning method with default ultrasonic velocity, the positioning accuracy is greatly improved. Compared with the ultrasonic positioning method with additional temperature compensation, the positioning accuracy obtained by the proposed method is comparable and has the advantage of no additional temperature sensors required. Compared with the general sensor configuration, the simplified sensor configuration is more advantageous and is recommended for practical applications.

No additional temperature sensors or calibration process are needed in the new proposed method. This new method does not directly rely on time-of-flights measurement to calculate the distance, but obtains the coordinates of the target according to the ratios of time-of-flights. In this way, the influence of the environmental temperature on positioning is avoided. This work provides a new approach for high accuracy ultrasonic positioning of the autonomous mobile robot based on multiple ultrasonic sensors.

In this work, a new ultrasonic positioning method using the ratios of time-of-flights obtained from multiple ultrasonic sensors is proposed and the point target is the main focus. The feasibility and effectiveness of the proposed method are verified by practical positioning experiments. Useful knowledge and experience reference of positioning with multiple ultrasonic sensors have been obtained. The research results can provide a useful reference for others’ research work and may have wide potential applications in many aspects of autonomous mobile robot, such as collision avoidance, guidance, navigation, mapping, target classification, and so forth. However, due to the complexity of positioning, more research work should be undertaken in future. To study the influence of configuration parameters on the positioning error and to realize the positioning of more complex cases (including the targets with complex shapes or 3D targets) will be our further research work.

## Figures and Tables

**Table 1:** Experimental results with the generalized measurement model.

		1	2	3	4	5	6	7	8	9
**S1**	$l_{r1}$ **(mm)**	244.0	267.0	291.0	315.0	363.0	411.0	460.0	509.0	558.0
	$l_{d1}$ **(mm)**	241.0	262.0	285.0	308.0	357.0	402.0	450.0	497.0	546.0
	$l_{c1}$ **(mm)**	245.3	266.7	290.1	313.6	363.4	409.2	458.1	506.0	555.8
	$l_{m1}$ **(mm)**	244.4	268.8	289.4	314.9	364.5	406.7	462.6	506.1	560.2
	**$e_{l_{d1}}$**	1.23%	1.87%	2.06%	2.22%	1.65%	2.19%	2.17%	2.36%	2.15%
	**$e_{l_{c1}}$**	0.55%	0.10%	0.30%	0.46%	0.12%	0.43%	0.41%	0.60%	0.39%
	**$e_{l_{m1}}$**	0.15%	0.68%	0.54%	0.02%	0.40%	1.05%	0.56%	0.57%	0.39%
**S2**	$l_{r2}$ **(mm)**	175.0	200.0	225.0	250.0	300.0	350.0	400.0	450.0	500.0
	$l_{d2}$ **(mm)**	173.0	197.0	220.0	246.0	295.0	343.0	393.0	441.0	490.0
	$l_{c2}$ **(mm)**	176.1	200.6	224.0	250.4	300.3	349.2	400.1	448.9	498.8
	$l_{m2}$ **(mm)**	175.4	202.1	223.4	251.5	301.2	347.0	404.0	449.1	502.7
	**$e_{l_{d2}}$**	1.14%	1.50%	2.22%	1.60%	1.67%	2.00%	1.75%	2.00%	2.00%
	**$e_{l_{c2}}$**	0.64%	0.28%	0.46%	0.17%	0.11%	0.23%	0.02%	0.23%	0.23%
	**$e_{l_{m2}}$**	0.23%	1.06%	0.70%	0.61%	0.39%	0.86%	1.00%	0.21%	0.55%
**S3**	$l_{r3}$ **(mm)**	233.0	255.0	278.0	301.0	348.0	396.0	444.0	492.0	541.0
	$l_{d3}$ **(mm)**	230.0	251.0	272.0	297.0	341.0	390.0	437.0	485.0	531.0
	$l_{c3}$ **(mm)**	234.1	255.5	276.9	302.4	347.1	397.0	444.9	493.7	540.6
	$l_{m3}$ **(mm)**	233.2	257.5	276.2	303.7	348.1	394.5	449.2	493.9	544.8
	**$e_{l_{d3}}$**	1.29%	1.57%	2.16%	1.33%	2.01%	1.52%	1.58%	1.42%	1.85%
	**$e_{l_{c3}}$**	0.49%	0.21%	0.39%	0.45%	0.25%	0.26%	0.20%	0.35%	0.08%
	**$e_{l_{m3}}$**	0.09%	0.99%	0.64%	0.89%	0.03%	0.37%	1.18%	0.38%	0.70%

**Table 2:** Experimental results with the simplified measurement model.

		1	2	3	4	5	6	7	8	9
**S1**	$l_{r1}$ **(mm)**	199.0	221.0	244.0	267.0	315.0	363.0	411.0	460.0	509.0
	$l_{d1}$ **(mm)**	196.0	217.0	242.0	263.0	309.0	356.0	401.0	451.0	500.0
	$l_{c1}$ **(mm)**	199.5	220.9	246.4	267.7	314.6	362.4	408.2	459.1	509.0
	$l_{m1}$ **(mm)**	199.1	220.7	245.1	267.1	317.5	362.5	414.3	457.9	503.3
	**$e_{l_{d1}}$**	1.51%	1.81%	0.82%	1.50%	1.90%	1.93%	2.43%	1.96%	1.77%
	**$e_{l_{c1}}$**	0.27%	0.04%	0.97%	0.28%	0.14%	0.16%	0.67%	0.19%	0.00%
	**$e_{l_{m1}}$**	0.05%	0.14%	0.46%	0.03%	0.79%	0.15%	0.80%	0.46%	1.12%
**S2**	$l_{r2}$ **(mm)**	175.0	200.0	225.0	250.0	300.0	350.0	400.0	450.0	500.0
	$l_{d2}$ **(mm)**	172.0	196.0	222.0	246.0	295.0	343.0	391.0	442.0	491.0
	$l_{c2}$ **(mm)**	175.1	199.5	226.0	250.4	300.3	349.2	398.0	450.0	499.9
	$l_{m2}$ **(mm)**	174.7	199.3	224.9	249.8	303.1	349.2	404.0	448.7	494.2
	**$e_{l_{d2}}$**	1.71%	2.00%	1.33%	1.60%	1.67%	2.00%	2.25%	1.78%	1.80%
	**$e_{l_{c2}}$**	0.06%	0.23%	0.45%	0.17%	0.11%	0.23%	0.49%	0.01%	0.03%
	**$e_{l_{m2}}$**	0.16%	0.33%	0.06%	0.07%	1.03%	0.22%	0.99%	0.28%	1.15%
**S3**	$l_{r3}$ **(mm)**	207.0	228.0	250.0	273.0	320.0	367.0	415.0	463.0	512.0
	$l_{d3}$ **(mm)**	203.0	224.0	246.0	269.0	314.0	359.0	406.0	456.0	503.0
	$l_{c3}$ **(mm)**	206.7	228.0	250.4	273.8	319.7	365.5	413.3	464.2	512.1
	$l_{m3}$ **(mm)**	206.2	227.8	249.2	273.2	322.6	365.5	419.5	463.0	506.3
	**$e_{l_{d3}}$**	1.93%	1.75%	1.60%	1.47%	1.88%	2.18%	2.17%	1.51%	1.76%
	**$e_{l_{c3}}$**	0.16%	0.02%	0.17%	0.31%	0.11%	0.42%	0.41%	0.26%	0.01%
	**$e_{l_{m3}}$**	0.38%	0.08%	0.33%	0.07%	0.82%	0.41%	1.08%	0.01%	1.11%
